# Warning: Anti-tobacco activism may be hazardous to epidemiologic science

**DOI:** 10.1186/1742-5573-4-13

**Published:** 2007-10-22

**Authors:** Carl V Phillips

**Affiliations:** 1 University of Alberta School of Public Health, Edmonton, AB T6G 2L9, Canada

## Abstract

This commentary accompanies two articles submitted to *Epidemiologic Perspectives & Innovations *in response to a call for papers about threats to epidemiology or epidemiologists from organized political interests. Contrary to our expectations, we received no submissions that described threats from industry or government; all were about threats from anti-tobacco activists. The two we published, by James E. Enstrom and Michael Siegel, both deal with the issue of environmental tobacco smoke. This commentary adds a third story of attacks on legitimate science by anti-tobacco activists, the author's own experience. These stories suggest a willingness of influential anti-tobacco activists, including academics, to hurt legitimate scientists and turn epidemiology into junk science in order to further their agendas. The willingness of epidemiologists to embrace such anti-scientific influences bodes ill for the field's reputation as a legitimate science.

## Introduction

When *Epidemiologic Perspectives & Innovations *called for submissions that analyzed cases of abuse of epidemiology or epidemiologists by organized political interests, we expected to discover stories about industry and government, the entities most typically associated with using their power to the detriment of science. We did indeed learn of several cases where the organized interests abusing epidemiology were industry or government, and communicated with some of the researchers involved. But these stories had already appeared in the literature (there was one exception where the researchers were not yet ready to go public). It turned out that all of the submissions we received about stories that had not previously appeared in the literature involved attacks on epidemiologists or epidemiology by anti-tobacco activists. Two of those submissions (both of which relate to the effects of passive exposure to cigarette smoke (a.k.a. "second-hand smoke" or environmental tobacco smoke (ETS)) appear with this commentary.

This outcome was particularly striking to me, since between the time of the first call for papers and this publication, I too became the target of abuse by anti-tobacco activists. This coincidence allows me to offer further information and insight in this commentary.

## Discussion

The accompanying article by Michael Siegel [1] recounts a particularly egregious misrepresentation of epidemiologic research by self-styled public health advocates. This might not be considered newsworthy, except for the fact that Siegel – a respected researcher and writer, and well-known anti-smoking advocate in his own right – actively attempted to correct the misrepresentation and was attacked for this. Indeed, Siegel's defenses of epidemiologic evidence, which anti-tobacco advocates preferred to ignore or misrepresent, resulted in him being "excommunicated" (there is really no other word that captures it) from the anti-smoking activists' inner circles.

The situation described in the article by James Enstrom [2] has gone even further, representing not only a bastardization of epidemiologic research by anti-tobacco advocates and an excommunication of a long-time member of the anti-smoking research club, but a concerted effort by political activists to destroy the career of a scientist because of one result that appeared in his data, which he chose to publish rather than suppress or alter to be more politically correct.

The three cases (including my own experience, described below) involve scientists whose careers are substantially devoted to the anti-smoking cause, but who have been viciously attacked by anti-tobacco advocates for not adhering 100% to the party line. These attacks are examples of the threat to honest science by powerful organized interests, a threat to which the science of epidemiology seems particularly vulnerable [3,4].

Enstrom's story of a fierce fight surrounding the result of an epidemiologic study is rather incongruous in the field of epidemiology, where most results are blindly accepted in spite of major limitations of the methods (much to the detriment of the science). Closer examination shows a pattern that is more familiar in public health than in legitimate scientific debate: There was no serious debate about the science. The predominant effort by those who attacked Enstrom and his study was an *ad hominem *smear campaign. Alas, all the energy that went into fighting about an epidemiologic result contributed absolutely nothing to improving the conduct of our shaky science. Readers of Enstrom's article may find it unusual to see an article that names names and makes clear statements about poor scientific conduct. But the careful reader will find that any shock should be directed not at the author who wrote this or the journal that published it, but rather at those whose actions undermined the scientific integrity of epidemiology and forced him to write it in the first place.

Readers of the Siegel article may find his story less surprising, given how common it is for activists of various stripes to casually misconstrue epidemiologic findings to support their political ends. But the full story is really more disturbing than that: Almost no other researchers have joined Siegel in pointing out the errors in the claims he brought to light, even though the claims in question were not remotely plausible. Moreover, respected organizations that are the face of epidemiology to most of the public (in particular, the major player of the Enstrom story, the American Cancer Society (ACS)), organizations that claim scientific authority, have joined the chorus that makes the ridiculous claim that Siegel has critiqued. (Several new chapters of this story have been written since Siegel finalized this manuscript [5-7], and they can be found in his blog [8], which is the best source of honest, scientifically-sophisticated, up-to-date analysis on matters related to tobacco policy.)

### ETS as a case study in junk science

Attempts to misrepresent the ETS literature are particularly disturbing given that the activists' primary goal seems to be to convince people that smoking is unhealthy, about the easiest argument to make based on an honest interpretation of the epidemiology. There is little doubt that inhaling smoke is unhealthy, but equally clear evidence shows that we can only demonstrate disease risk from ETS for those at the highest level of exposure. The evidence about health effects of smoke and the legitimate aesthetic objection to involuntary ETS exposure are quite sufficient to justify prohibiting indoor smoking in public places, though clearly insufficient to justify public policies that prohibit voluntary low-level ETS exposure. Fighting over the details, then, either has no practical implications or is intended to promote policies that are not warranted by public health concerns, suggesting that those who misrepresent the epidemiology are willing to undermine science for rather trivial gain. The activists involved, many of whom hold titles that indicate they should behave as scientists and academics, appear unconcerned about subverting science to further their worldly agendas, hurting the careers of honest scientists, driving students away from politically controversial fields, attacking the principles of free academic research, and threatening the reputation of epidemiology as a field.

Readers interested in further distortions of science relating to ETS research should read Siegel's blog entries that mention the Helena, Montana study or the recent reports from New York. It was claimed by authors who positioned themselves as epidemiologists that ETS exposure in public places causes 10%, or perhaps 40%, or maybe even 60% of all heart attacks. It is difficult to understand how such patently absurd claims can be made without an outcry from legitimate scientists.

### An additional example of the threats to legitimate research

My own situation further demonstrates the threat that anti-tobacco activists pose to legitimate epidemiology. Much of my work focuses on "tobacco harm reduction", the possibility that smokers who will not give up nicotine might be convinced to switch to smokeless tobacco [9,10]. Researchers and practitioners who are concerned with reducing the health impact of smoking are increasingly embracing this strategy. Smokeless tobacco provides nicotine at a level and rate that can be satisfying to smokers (in contrast with the popular pharmaceutical nicotine products), overwhelming evidence shows it is about 99% less harmful than smoking [11], and the experience in Sweden and Norway shows that a large portion of smokers are willing to switch. Unfortunately, the anti-tobacco orthodoxy has chosen to condemn and oppose this approach in favor of the ineffective abstinence-only (a.k.a. "quit or die") strategy.

Challenges to tobacco harm reduction might represent a legitimate scientific or policy debate, were it not for the tactics employed. Those tactics take the form of ignoring the scientific and policy analysis evidence, publishing misinformation to mislead the public [12,13], and trying to censor those who support harm reduction. In my case, there has been a concerted effort by activists to shut down the research done by my research group at the University of Alberta School of Public Health, and terminate our employment.

The attacks on me have apparently been largely instigated by activists outside my university, though as happened with Enstrom, it was academics who were directly responsible for some of the most shameful action. In my case, faculty at the University of Alberta School of Public Health (UASPH) voted to forbid me from accepting the grant funding that supports my research group. This came despite the fact that my research focus and funding were carefully considered and approved at the department, faculty, and university levels when I was recruited to come to the University of Alberta (the UASPH was created subsequent to my arrival 2 1/2 years ago, and my appointment was moved there).

The UASPH administration sponsored the vote and actively advocated that the faculty vote against me; the core of their substantive argument was that outside complaints about the content of my research were posing a political problem for the school. As further evidence of the central role of political pressure, the UASPH administration talked to the press immediately after the vote to make sure the details of their action would be publicly reported the next day. In the weeks leading up to that vote, one of the top administrators at the UASPH declared that if I did not shift my research focus then he was not inclined to support my continued presence in the department (even though the department had already lost more than 1/3 of its faculty that year). Shortly after the vote to cut off my funding, the UASPH dean notified me that because I lacked funding (which was not actually true; I had various options for continued support), my position was being terminated. Shortly after that, the UASPH administration started communicating to my graduate students behind my back, suggesting to them that I was planning on abandoning them. I have also been subject to audits of my research account expenditures, during which the administration actually declared that they had a right to tell me I could not read certain books as part of my research. My trainees and other members of my research group have faced inappropriate scrutiny, interference, and accusations by the human subjects ethics review board, which is run by a professor who actively lobbied for the vote to cut my funding.

Fortunately, just before the time of this publication, following a front-page story about my situation in one of Canada's national newspapers, the National Post [14], my situation improved dramatically. The University of Alberta administration, which has consistently supported my academic freedom, explicitly communicated that I (and other professors) at the University are free to take research grants from any legal source (in my case, part of my funding comes from the smokeless tobacco industry) that does not come with unethical encumbrances (my funding is unrestricted: the funder has no influence over what I do with it and no access to the research until is published). In particular, the University of Alberta has made clear it is not succumbing to outside pressure to cut off all funding from the tobacco industry.

This principled stand by a university in support of academic freedom, while showing that reason often prevails, does not change the fact that anti-tobacco activists, including some who consider themselves academics, interfered with my research, cost me and my staff more than a person-year's worth of productivity, and misled the public. Nor does it change the pattern of what my colleagues and I have faced for doing research that defies the anti-tobacco orthodoxy, including just in the last year or so:

-*ad hominem *attacks on the legitimacy of my employment and research focus (though never actually on the substance of my work) in the local press (sometimes under the guise of news) by local activists and even professors at the University;

-vandalism of one of our posters at an academic conference;

-threats to one of my students regarding her internship and future employment prospects that resulted in her giving up co-authorship for legitimate research she had done;

-threats made by activists (that presumably motivated some of the aforementioned behavior by UASPH administrators) that they would try to prevent the UASPH from getting accreditation if I were allowed to keep doing my work there;

-not being allowed to participate in tobacco-related conferences, including a recent major Canadian semi-academic conference held in Edmonton, the "5^th ^National Conference on Tobacco or Health";

-forced cancellation due to blackmail of a conference on academic freedom and research integrity we organized to coincide with and respond to the aforementioned conference: the organizers of the aforementioned anti-tobacco conference threatened the conference center that they would cancel their much larger contract if we were allowed to hold our conference, and we agreed to let the conference center out of their contract with us rather than put them in the middle.

Enstrom found himself being, in effect, named as a racketeer due to the actions of anti-tobacco activists because of one study result he published. Ironically, the racketeering law under which he was implicitly accused was designed to punish cabals that use threats and intimidation to try to influence the legal behavior of others.

### Money, power, and the funding smokescreen

Many of the attacks against Enstrom and me, though clearly directed at the content of our research, have been rationalized based on it being partially funded by unrestricted grants from the tobacco industry. The evidence that this is a rationalization can be found in the similarly vehement attacks on those who defied the orthodoxy but have not received industry funding, including Siegel. Enstrom cites several studies that produced results similar to his and that were not supported by industry grants, but his antagonists paid them no more attention than they did his work.

Moreover, much of the funding from anti-tobacco organizations, both government and private, comes with major strings attached, often all but declaring what the conclusions of the research should be. So it is clear that these organizations do not actually have a deep-seated concern about the influence of funding. Hardly a word is heard from that quarter about the pharmaceutical industry or others who have a financial interest in tobacco use or cessation methods, and who help fund the anti-tobacco organizations. Rather, those organizations are intent on making sure that they get to control the funding, and thus the agenda, in "their" area, and the only significant threat to this monopoly is tobacco industry funding. For example, despite the fact that anti-tobacco organizations' funds dwarf tobacco industry grants to academic researchers, no major research effort in tobacco harm reduction has been able to get substantial funding without seeking it from the industry.

Anti-tobacco activists have long coasted on the cigarette industry's misdeeds regarding producing illegitimate research. The substantial and deplorable misdeeds from thirty or forty years ago are well documented, and it is clear that the industry has been guilty of many of the same crimes against epidemiology practiced by anti-tobacco activists today. One result of that guilt coming to light is that claims by the industry are widely discounted, making them little present threat to honest science. Despite this, anti-tobacco activists still try to attribute epidemiology that they do not like to the (largely nonexistent) influence of the industry in the field. Another result of the guilt is that the industry's every move is carefully watched, making tobacco industry funding a professor's dream: the funder does not dare say a word to try to influence the research.

Indeed, in our society today, it is difficult to imagine for-profit corporate entities thinking they could get away with actions like those taken by anti-tobacco activists. There is no doubt that powerful, rich organizations can be a threat to good science, and in the case of tobacco research, it is the multi-billion dollar anti-tobacco industry that currently plays that role.

### Turning epidemiology into junk science?

Epidemiology is often dismissed as junk science. There are many who seem willing to make it so, and few who actively defend against this tendency.

Researchers with political agendas often seem willing to bias how they interpret their data to better support their worldly goals. Researchers who work as part of the anti-tobacco orthodoxy appear particularly willing to do so. Selective citation and cherry-picking favored results is woefully common, but it is difficult to think of a case as bad as the one Enstrom reports, in which the chief *epidemiologist *for the ACS vehemently accuses someone else of bias on a topic while conveniently ignoring his own organization's data and a dissertation he advised. (I found this less surprising than others might, given that the ACS also continues to claim in its public pronouncements that smokeless tobacco poses a major risk for oral cancer, despite the fact that their own research studies are part of the overwhelming evidence that it does not [15,16].)

Enstrom points out that biased analysis in the form of selective citation and "publication bias *in situ*" [17] should be considered a serious ethical violation. The defamatory attacks on him are certainly the most egregious acts in the story. The dismissal of sound science and attempts to intimidate honest researchers into adhering to the preferences of powerful organizations are major threats to the science. But it is probably the commonness of biased analysis, presentation, and citation of results that poses the greatest total threat to epidemiology as a science.

Figuring out how epidemiology can police itself against manipulation in support of authors' advocacy goals is a critical challenge for the field; it is not just an ethical necessity, but also a critical tactic in attempting to gain credibility and influence. A few years from now, when it is obvious to the public and policy makers that a substantial portion of the epidemiologic claims they heard for years (and that passed without challenge) were garbage, it will be an easy victory for those who wish to tar all of epidemiology with the label junk science.

The work of Enstrom and Siegel demonstrates the difficulty that good epidemiologists have in disputing well-funded public health propaganda, or even mere sloppy science. So long as there is no penalty for promulgating junk epidemiology, whether it is junk science by intention or simply due to poor methodology, all epidemiology will remain suspect. Efforts like Siegel's blog and Enstrom's digging into the ETS literature are rare, and a few people cannot possibly hope to keep up with a deluge of weak science and overt propaganda. The war against junk science cannot be won by junk hauling alone. This is especially true when *ad hominem *attacks are not soundly denounced by everyone in the field, even by those who disagree with the scientific claims of those being attacked.

Despite attempts to carefully hunt down hidden information (e.g., the Cardenas dissertation or the ETS covariate in the air pollution study) or re-analyze data and results (e.g., Enstrom and Kabat's meta-analysis that corrected the errors in Glantz's and provided a much more transparent and complete publication), most people and most epidemiologists probably still believe that mortality from ETS has been shown to be high. While a major share of the blame for this certainly goes to the massive anti-tobacco industry's propaganda, such propaganda would not get any traction if the standards of epidemiology did not facilitate such misuse of data.

Those who try to misconstrue scientific evidence to deny Darwinian evolution have big budgets and loud voices too, but gain little traction outside of their core constituencies. The difference seems to be that evolutionary biology is sufficiently robust as a scientific field that most moderately educated people can sort the science from the silliness with a modest amount of effort. Biologists and other scientists whose work has little to do with evolution speak up in defense of the theory of evolution when it is attacked based on religious creation myths. But when core values and principles of epidemiology are attacked by quasi-religious zealots, many epidemiologists seem quite willing to join the zealots. There is certainly no united front in defense of the science.

One possible interpretation of this is that there is no actual science of epidemiology, there is just a bunch of people using a collection of methods to analyze certain types of data (a hot-button characterization in the field, but one that presumably touches a nerve because it strikes close). A better interpretation seems to be that while there are real scientists and a real science, there are just too many non-scientists – as defined by fundamental attitudes toward inquiry, rather than education or practice – who practice epidemiology. I recently argued that epidemiology suffers so much from outside influence, particularly from the agendas of self-styled defenders of public health, because it lacks the scientific *gravitas *to withstand such influences [3].

## Conclusion

Enstrom cites the reign of terror over biology under Stalin as one example of politics trumping science. Though the Soviet case is rather extreme (we North Americans who dare question the scientific orthodoxy only have our careers threatened; not our lives, at least so far), it is not the most extreme. Many cultures were hobbled for centuries because of religious adherence to pseudoscience, and damage to people's health was one of the many results.

To conclude, I will offer a footnote to the Enstrom story, related to the session he hosted at the Congress of Epidemiology/Society for Epidemiologic Research, "Reassessment of the Long-term Mortality Risks of Active and Passive Smoking." Enstrom was not aware, at the time he wrote his article, that Jonathan Samet suggested to conference participants that they boycott (Samet's own word) that session. While this is hardly startling when mentioned at the end of a series of papers that describe exclusion, censorship, blackballing, and blackmail by the anti-tobacco establishment in their attempts to stifle dissent, its implications are darker than they seem at first blush: This was a real scientific meeting, not an anti-tobacco conference. A call for a boycott is not merely speaking ill of a researcher or study (time-honored traditions in science); it is a suggestion that others avoid even listening to presentations of evidence and analysis that those in power do not like. This is not legitimate scientific argument, or even a mere petulant protest. It is an attempt to promote the kind of self-censorship of thought examined by Orwell and mastered by Stalin. This took place at a premier scientific meeting in the field of epidemiology, and yet the suggestion did not appear to be denounced by anyone. This suggests that epidemiologists lack respect for their field as a legitimate science, and accept its role as a tool to be manipulated for advocacy, an attitude which seems attributable in no small measure to anti-tobacco activism and similar forms of advocacy.

## Abbreviations

ACS: American Cancer Society

ETS: Environmental tobacco smoke (a.k.a. second-hand smoke)

UASPH: University of Alberta School of Public Health

## Competing interests

The author's interests that are potentially furthered by writing this paper include those that are implicitly presented in the manuscript, specifically: an interest in keeping epidemiology from being junk science; an interest in actively defending legitimate science and scientists; an interest in making *Epidemiologic Perspectives & Innovations *play an important role in improving the field; an interest in promoting public health by communicating that smokeless tobacco is a reduced-harm alternative to smoking; and an interest in defending his ability to do research that refines our knowledge related to tobacco harm reduction (including  being able to get the industry grants that make it possible). The author's narrow self-interest – with regard to employment security, access to research grants, personal wealth, and generally making life easier – directly competes with these goals.
